# Vertical disease programs and their effect on integrated disease surveillance and response: perspectives of epidemiologists and surveillance officers in Nigeria

**DOI:** 10.1186/s40794-021-00152-4

**Published:** 2021-10-01

**Authors:** Francis Idenyi Onwe, Ijeoma Nkem Okedo-Alex, Ifeyinwa Chizoba Akamike, Dorothy Ogechi Igwe-Okomiso

**Affiliations:** 1 Ebonyi State Ministry of Health, Abakaliki, Nigeria; 2 Department of Community Medicine, Alex Ekwueme Federal University Teaching Hospital, Abakaliki, Ebonyi State Nigeria; 3 African Institute for Health Policy and Health Systems, Ebonyi State University, Abakaliki, Nigeria

**Keywords:** Integrated disease surveillance and response, Vertical disease programs, Disease surveillance, Nigeria, Donor funding

## Abstract

**Background:**

Integrated Disease Surveillance and Response (IDSR) is a cost-effective surveillance system designed to curb the inefficiency associated with vertical (disease-specific) programs. The study determined the existence and effect of vertical programs on disease surveillance and response in Nigeria.

**Methods:**

A cross-sectional study involving 14 State epidemiologists and Disease Notification Surveillance Officers (DSNOs) in 12 states located within the 6 geopolitical zones in Nigeria. Data was collected using mailed electronic semi-structured self-administered questionnaires. Response rate was 33.3%. The data was analyzed using SPSS version 20.

**Results:**

Half of the respondents were males (50.0%) and State epidemiologists (50.0%). Malaria, HIV/AIDS, tuberculosis, and other diseases were ongoing vertical programs in the States surveyed. In over 90% of cases, vertical programs had different personnel, communication channels and supportive supervision processes different from the IDSR system. Although less than 50% acknowledged the existence of a forum for data harmonization, this forum was ineffectively utilized in 83.3% of cases. Specific disease funding was higher than that of IDSR (92.9%) and only 42.9% reported funding for IDSR activities from development partners in the State. Poor data management, low priority on IDSR priority diseases, and donor-driven programming were major negative effects of vertical programs. Improved funding, political ownership, and integration were major recommendations preferred by the respondents.

**Conclusion:**

We found that vertical programs in the surveyed States in the Nigerian health system led to duplication of efforts, inequitable funding, and inefficiencies in surveillance. We recommend integration of existing vertical programs into the IDSR system, increased resource allocation, and political support to improve IDSR.

## Introduction

Programs with specific objectives, focused on specific health programs are in particular attractive to donors because of the need to have measurable investment results [1]. This describes the nature of vertical programs which usually have quantitative, specific, and defined objectives and typically target a single condition or small group of health problems. In addition, vertical programs have short or medium-term objectives with centralized management [1]. Some other advantages of vertical programs include financial control, the ability to respond to changing circumstances, and the identification of new strategies. The fact that these programs have objectives that are achievable in a limited time frame and are preferred by external donors makes them beneficial [1]. Other benefits include the ease of evaluation of single programs.

Although there are benefits of vertical programs, several challenges have been identified with vertical, single-disease surveillance strategies [2]. Vertical programs are expensive, with duplication of services. One main drawback is that most vertical programs are designed to merely provide data to central levels with little or no coordination between those collecting it, analyzing it or those using it for decision-making [2].

Despite these gaps, many intervention programs still rely on their own disease surveillance systems [3]. Consequently, the World Health Regional Committees for Africa advocated Integrated Disease Surveillance and Response (IDSR) in 1998 at its 48th session with the aim of integrating multiple surveillance systems [4]. Integration ensures that human and other resources are used more efficiently and effectively [3].

Public health surveillance is the ongoing, systematic collection, analysis, interpretation, and dissemination of data about a health-related event for use in public health action to reduce morbidity and mortality and to improve health [3, 5]. The various functions of surveillance include supporting case detection and public health interventions, estimating the impact of a disease or injury, portraying the natural history of a health condition, determining the distribution and spread of illness, generating hypotheses and stimulating research, evaluating prevention and control measures, and facilitating planning [5].

The IDSR is a strategy and a tool to promote rational use of resources by integrating and streamlining common surveillance activities [3]. In an integrated system, surveillance activities are coordinated and streamlined. Scarce resources are combined to collect information from a single focal point at each level instead of using the resources for separate vertical activities. Several activities are combined into one integrated activity and similar surveillance functions, skills, and resources are harnessed [3]. Surveillance and notification of diseases in Nigeria entails the immediate notification of epidemic-prone diseases, targeting of diseases for elimination and eradication, and monthly notification of diseases of public health importance [6]. The actual implementation of IDSR is important given the high burden of these diseases. For instance, in 2019, Nigeria had the highest number of malaria cases (25% of global malaria cases) and deaths globally (24% of global malaria deaths) [7]. These diseases are mostly controlled using vertical control and elimination programs. Additionally, Nigeria is one of the high tuberculosis (TB) burden countries globally ranking 6th among the 30 high TB burden countries in 2019 [8]. Nigeria has experienced several outbreaks such as the yellow fever outbreak in 1986 and 1987 [9, 10], meningitis outbreak in 1996 and 2017 [11, 12], cholera outbreaks in 2001 and 2004 [13], and Ebola virus disease outbreak in 2014 [14]. Others include Lassa fever, monkey pox epidemics and the COVID-19 pandemic [15–17].

It has been shown that the shortcomings of the vertical disease surveillance strategies have not all equally been successfully overcome by IDSR [18]. A systematic review of the challenges with implementation of the IDSR in low-and-middle-income countries was carried out in 2012 and revealed that numerous systems with unique reporting requirements still persist. Some of the challenges include non-sustainable financial resource strategies, inadequate training, lack of supervision from higher authorities, and weak laboratory capacities. Inadequate job aids such as case definitions, reporting formats, and poor communication and transport systems were also listed as challenges [18]. It is therefore important to review the existence of vertical programs and understand the magnitude of their effect on IDSR. This will help in providing evidence-based recommendations on how to improve the surveillance system. The objective of this study was to determine the existence and effect of vertical programs on disease surveillance and response in Nigeria.

## Methods

### Study setting and design

This study was conducted in Nigeria. Nigeria is located in West Africa within sub-Saharan Africa. Nigeria has a total land area of 923,768 km^2^ and is the most populous nation in Africa with a population of approximately 180 million people [19]. Nigeria has 36 States and 6 geopolitical zones namely South-East, South-West South-South, North-Central, North-West and North-East. Each geopolitical zone has 5–7 States under it. It has about 374 ethnic groups with Igbo, Hausa, and Yoruba as the major ones.

The country’s health system has 3 tiers: federal, state, and local government levels and health is on the concurrent list meaning that each level is responsible for the health system at its level. Nigeria adopted the IDSR strategy in 2000 replacing the Disease Surveillance and Notification (DSN) system. The State ministry of health (SMOH) is responsible for the health system at the State level. The epidemiology unit within the SMOH headed by the State epidemiologist oversees IDSR activities and forms a crucial component of the flow of surveillance information from the local to the federal government. The other key stakeholder in the epidemiology unit is the State Disease Notification Surveillance Officer (DSNO) who coordinates the activities of the local government DSNOs towards timely, quality, effective and efficient surveillance for priority diseases.

This study had a cross-sectional design and was conducted among State epidemiologists and Disease Notification Surveillance Officers in 12 States of Nigeria.

### Study population and sampling

The study population were State epidemiologists and DSNOs in ministries of health in Nigeria. Those who did not give informed consent to participate in the study were excluded. The participants were purposively selected across States in all the geopolitical zones of Nigeria.

### Data collection

The study was conducted in February 2018. Data was collected using mailed electronic semi-structured self-administered questionnaires. (Response rate = 33.3%). Weekly email reminders were sent to the participants over the 4 weeks. The questionnaire was designed following a review of existing literature and based on contextual work experience in Ebonyi State epidemiology unit. Socio-demographic and work-related information was collected in the first section of the questionnaire. The second section of the questionnaire was on the existence of vertical programs and assessed the data management, personnel funding, and effects of vertical programs compared to IDSR. Thirteen Yes/No questions were used for this section. The third section sought information on funding and equipping of IDSR and the epidemiology unit in the various States. This was assessed using four Yes/No questions.

The last section reviewed the effects of vertical programs on IDSR and the proposed recommendations to improving the IDSR program using two open-ended questions.

### Data analysis

Data were analyzed using the Statistical Package for Social Sciences (IBM-SPSS) for Microsoft Window version 20 software. Frequencies and proportions were calculated and results were presented using tables. The responses to the open-ended questions were summarized in a table using the health system building blocks as a guide for the categorization. Other emergent categories were also highlighted.

## Results

The respondents were equally distributed by gender (males = 50.0%) and designation (State epidemiologists = 50.0%). About half (42.9%) were from the South-South geopolitical zone. Most of the respondents had served for 1-3 years in their designated position in the state epidemiology unit (Table 1).

**Table 1:** Socio-demographic characteristics of the respondents

Variable	Frequency (n)	Percent (%)
**Gender**		
Male	7	50
Female	7	50
**Geopolitical zone**		
South East	1	7.1
South west	2	14.3
South-South	6	42.9
North Central	1	7.1
North West	2	14.3
North East	2	14.3
**Designation**		
Epidemiologist	7	50
Disease Surveillance and Notification Officer	7	50
**Duration in Position**		
1–3 years	8	57.1
4–6 years	3	21.4
7–9	1	7.1
> 10 years	2	14.3

All respondents (100%) reported the existence of vertical programs in their respective States and agreed to the existence of vertical programs on Malaria, HIV/AIDS, tuberculosis, and Neglected Tropical Diseases in their States. Most of them stated that vertical programs have different data forms from IDSR (78.6%) and that these data did not correspond with that from IDSR (92.9%). The existence of a forum for data harmonization between IDSR and vertical programs was reported by 42.9% of the respondents with 83.3% of those who reported the existence of the form stating that it was not effectively utilized (Table 2).

**Table 2:** Existence of vertical disease programs and program data management in the States (*n* = 14)

Variable	Frequency	Percent (%)
**Existence of vertical programs**		
Yes	14	100
No	0	0
**Existing vertical programs**		
Malaria	14	100
HIV/AIDS	14	100
Tuberculosis	14	100
Neglected Tropical Diseases	14	100
Leprosy	13	92.9
Other diseases	6	42.9
**Vertical programs have different data forms from IDSR**		
Yes	11	78.6
No	3	21.4
**Vertical program data corresponds with IDSR data**		
Yes	1	7.1
No	13	92.9
**Presence of forum for data harmonization between IDSR and vertical programs**		
Yes	6	42.9
No	8	57.1
**Forum effectively utilized (*n*** **= 6)**		
Yes	1	16.7
No	5	83.3
**Communication channels for vertical programs same with that of IDSR**		
Yes	1	7.1
No	13	92.9

The majority (92.9%) reported that the personnel for data collection for vertical programs was different from that used for IDSR. There was inadequate IDSR funding and non-integrated supportive supervision involving the vertical program managers and the state epidemiology unit reported by 92.9% of the respondents. Most respondents (92.9%) agreed that partners selectively fund vertical programs with less funding for IDSR. Funding for the IDSR activities by development partners funding was reported by 42.9% of the respondents (Table 3).

**Table 3:** Funding of the state epidemiology unit and paraphernalia

Variable	Frequency	Percent (%)
**The state epidemiology unit has the following**		
Official vehicle	3	21.4
Generator	1	7.1
Office internet	2	14.3
Monthly imprest	3	21.4
**Vertical disease program equipment/infrastructure like vehicle, generator, internet etc. being used together with epidemiology unit**		
Yes	2	12.5
No	12	87.5
**if funds spent on vertical programs are collectively used for all the IDSR diseases and activities, surveillance performance and therefore disease prevention and control will greatly improve**		
Yes	14	100
No	0	0

Availability of official car and monthly imprest were each cited by 21.4% of the epidemiology units. Most respondents (87.5%) alluded that the epidemiology units do not use equipment together with vertical programs. All participants agreed that collective use of funds spent on vertical programs would improve surveillance and disease prevention (Table 4).

**Table 4:** Vertical Program and IDSR personnel, funding and management in the States (*n* = 14)

Variable	Frequency	Percent (%)
**Personnel for data collection for vertical programs same with that of IDSR**		
Yes	1	7.1
No	13	92.9
**Integrated supportive supervision involving the vertical program managers and the state epidemiology unit exists**		
Yes	1	7.1
No	13	92.9
**Development partners fund IDSR activities in your state**		
Yes	6	42.9
No	8	57.1
**How many apart from World Health Organization (*n*** **= 6)**		
0ne	2	33.3
Two	3	50.0
Three	1	16.7
**IDSR adequately funded in your state**		
Yes	1	7.1
No	13	92.9
**Partners selectively fund a specific disease (vertical program) and not IDSR**		
Yes	13	92.9
No	1	7.1
**Specific disease funding is higher than that of IDSR**		
Yes	13	92.9
No	1	7.1

The effects of vertical programs on IDSR stated by the participants include donor-driven funding and terms of reference, emphasis on service delivery rather than surveillance, and poor funding (Table 5).

**Table 5:** Effects of vertical programs on the IDSR program

Broad area affected	Instances
**Health information management**	Poor data management
	Different reporting mechanisms and data collection tools
	Data mostly on service delivery and not surveillance
**Program processes and structure**	Lack of linkages and synergies with other programs
	Surveillance is not stand-alone but is joined to Monitoring & Evaluation (M&E)
	Data is mostly on service delivery and not surveillance
	Celebration of WHO days for vertical program diseases and negligence for non-vertical program diseases
**Donor funding and influence**	Non-supported programs overburden the state government
	Donor advantage is more considered
	Diversion of supporting partners
	Donor driven funding
	Donor-specific terms of reference
**Funding**	Skewed and poor funding
	Multiplicity of funds
**Human resources**	Non-integrated personnel
	Terms of references for vertical program not well understood by the program officers
	Vertical program officers feel threatened by surveillance officers on whom they claim superiority due to funding allocated to them by partners

Integration of vertical programs into IDSR, improved funding, and equipping of IDSR, and capacity building of IDSR staff were some of the recommendations for improving the IDSR program (Table 6).

**Table 6:** Recommendations on how to improve the IDSR program by the respondents (*n* = 14)

Broad area	Instances
**Health information management**	Quarterly functional disease data consultative fora
	Provision of surveillance data tools
	One health (human and zoonotic) disease data capturing
	Harmonize all reporting forms and channels of reporting
**Program governance, processes and structure**	All disease programs must be mandated to report through and be collapsed into IDSR
	High level advocacy
**Donor funding and influence**	Development partners should not undermine one program for another
**Funding**	Basket funding
	Improved funding and equipping of IDSR desk officers are the way vertical disease program managers are funded and equipped,
**Human resources**	Capacity building and incentives to surveillance personnel
	Increased collaboration between epidemiologists and vertical program officers
	Supportive supervision for IDSR personnel

## Discussion

Despite the implementation process of IDSR in Nigeria since 2000 [20], this study has shown that there is a strong existence of vertical programs on diseases like malaria, tuberculosis, HIV, and NTDs in Nigeria. From our findings, almost all the vertical programs have different reporting forms from the available IDSR system. This resulted in ineffective utilization of data harmonization forums and multiple data reporting.

The study revealed that resources for these vertical programs were separate from those for IDSR including personnel, funding, and materials. This defeats the objective of the IDSR and consequently leads to a waste of resources [3].

This study also found that developmental partners funded more vertical programs than IDSR. Another finding from this study showed non-integrated supportive supervision involving the vertical programs managers and the state epidemiology unit. These findings are peculiar with donor-funded vertical programs [2, 21].

The major effects from the existence of these vertical programs included the following broad areas: Health information management; Program processes and structure; Donor funding and influence; and Human resources.

### Health information management

There was gross poor data management with different reporting mechanisms and data collection tools from the IDSR tools. This defeats the aim of the adopted IDSR. This could also result in poor response to outbreaks without an integrated surveillance system. Also, data reported were mostly on service delivery of the vertical programs and not on surveillance. This is similar to findings in a systematic review in LMIC [18] despite efforts by member states to integrate surveillance for efficiency.

The study participants recommended the following: quarterly functional disease data consultative fora, provision of surveillance data tools, one health (human and zoonotic) disease data capturing, and harmonization of all reporting forms and channels of reporting. Some studies have also recommended an integrated one health disease data capturing [22, 23] and harmonization of reporting channels [24] especially in the advent of emerging and re-emerging diseases like Ebola, brucellosis, dengue, and recently COVID 19.

Surveillance and responding to infectious disease outbreaks has been a major public health challenge in Nigeria, given its rapid population growth, increasing movement of people and destruction of infrastructure following the insecurity that has plagued the country in the recent past as well as outbreaks from new and re-emerging pathogens.

### Program processes and structure

The vertical programs lacked linkages and synergy and high-level advocacy was recommended for addressing this. All disease programs should be mandated to report through and be collapsed into IDSR. The onus lies on the national health system governance to ensure the integration and coordination of these programs in line with the country’s policy direction. A review on health system strengthening [25] also revealed that good governance using a multi-stakeholder approach is an important factor in improving health services and outcomes.

### Donor funding and influence

Vertical programs development partners fund these programs with no support for already existing programs and this comes with donor-specific terms of references. There is expected to be a pool of funds for integrated disease surveillance and response instead of the reported multiplicity of funds from vertical programs. Donor influence and lack of coordination by the government creates this diversion. There is eventually skewed and poor funding of IDSR programs. This finding is corroborated by a systematic review done in 2015 [18].

It is recommended that development partners should not undermine one program for another. Better country ownership and oversight of development partners with more intra and intersectoral collaboration (One health) can also further strengthen integrated disease surveillance. Basket funding has been recommended in health programs [26]. Improved funding and equipping of IDSR desk officers is also advocated. Again good governance is required here for implementation.

### Human resources

This study showed a non-integration and lack of cooperation and coordination of personnel in disease surveillance and response. Vertical program officers feel threatened by surveillance officers over whom they claim superiority due to funding allocated to them by partners. This consequently places a low priority on the IDSR priority diseases.

Capacity building and incentives to surveillance personnel is recommended, with increased collaboration between epidemiologists and vertical program officers. A similar recommendation was made in several studies study [21, 27, 28].

Vertical programs affect pillars of the health systems as evident above. Efforts have been made in Nigeria to strengthen the health system through strategies like the National Strategic Health Development Plan (NSHDP) II framework [26] which was founded on the eight pillars of the health system. A cohesive implementation framework for the NSHDP II was validated by stakeholders in 2017 [29]. This comprises the integration of health services including surveillance of diseases. A lot of effort on the part of leadership and governance is needed for the implementation of health programs including IDSR. Continuous involvement of stakeholders including leadership in the implementation of IDSR is necessary.

A rapid assessment of IDSR performance in 47 African countries between 2014 and 2017 showed that 44 of those countries (98%) were implementing IDSR though the quality of the implementation was not assessed [30]. The study revealed that Nigeria was among the few countries with less than 50 % coverage of IDSR at the subnational level as compared with other African countries with over 90 % coverage like Uganda, Rwanda, Liberia, Senegal and Togo [30]. This low coverage in Nigeria could be due to the vertical program’s surveillance running concurrently with IDSR.

Adoption of the National Technical Guidelines on IDSR has helped some countries in the management of recent outbreaks like Ebola [28, 31]. There is therefore a need for African countries and other LMICs to fully adopt their level National Technical Guidelines on IDSR and other integrated surveillance systems to be better positioned to prepare for and identify outbreaks like Ebola and COVID-19.

This study is not without limitations. Firstly, it only involved a limited number of epidemiologists with over and under-representation of some geopolitical zones thus its findings may not be generalizable to the whole of the country. However, the inclusion of epidemiologists from all geopolitical zones of Nigeria improves its representativeness. As is peculiar to the diversity of the Nigerian context, the States in the different geopolitical zones may have differences in demographic characteristics but they share similar IDSR structures across States. Because the study was based on self-reports, desirability bias may have played a role however this is unlikely because most of our findings have been associated with vertical programs. The additional use of observational checklists is recommended for future studies.

## Conclusion

This study found that the continuance of vertical programs in the Nigerian health system led to duplication of efforts, inequitable funding, and inefficiencies in surveillance. The authors therefore recommend the integration of existing vertical programs into the IDSR system, increased resource allocation, and political support to improve IDSR.

## Data Availability

The datasets used and/or analyzed during the current study are available from the corresponding author on reasonable request.

## Abbreviations

DSN: Disease Notification Surveillance
DSNO: Disease Notification Surveillance Officer
HIV: Human Immunodeficiency Virus
IDSR: Integrated Disease Surveillance and Response
NTD: Neglected Tropical Disease
NSHDP: National Strategic Health Development Plan
SD: Standard Deviation
SMOH: State Ministry of Health
SPSS: Statistical Package for Social Sciences
WHO: World Health Organization
